# canEvolve: A Web Portal for Integrative Oncogenomics

**DOI:** 10.1371/journal.pone.0056228

**Published:** 2013-02-13

**Authors:** Mehmet Kemal Samur, Zhenyu Yan, Xujun Wang, Qingyi Cao, Nikhil C. Munshi, Cheng Li, Parantu K. Shah

**Affiliations:** 1 Department of Biostatistics and Computational Biology, Dana-Farber Cancer Institute and Harvard School of Public Health, Boston, Massachusetts, United States of America; 2 Department of Biostatistics and Medical Informatics, Akdeniz University, Antalya, Turkey; 3 Department of Bioinformatics, School of Life Science and Technology, Tongji University, Shanghai, China; 4 State Key Laboratory for Diagnosis and Treatment of Infectious Diseases, The First Affiliated Hospital, School of Medicine, Zhejiang University, Hangzhou, China; 5 Department of Medical Oncology, Dana-Farber Cancer Institute and Harvard Medical School, VA Boston Healthcare System, Boston, Massachusetts, United States of America; King Faisal Specialist Hospital and Research Centre, Saudi Arabia

## Abstract

**Background & Objective:**

Genome-wide profiles of tumors obtained using functional genomics platforms are being deposited to the public repositories at an astronomical scale, as a result of focused efforts by individual laboratories and large projects such as the Cancer Genome Atlas (TCGA) and the International Cancer Genome Consortium. Consequently, there is an urgent need for reliable tools that integrate and interpret these data in light of current knowledge and disseminate results to biomedical researchers in a user-friendly manner. We have built the canEvolve web portal to meet this need.

**Results:**

canEvolve query functionalities are designed to fulfill most frequent analysis needs of cancer researchers with a view to generate novel hypotheses. canEvolve stores gene, microRNA (miRNA) and protein expression profiles, copy number alterations for multiple cancer types, and protein-protein interaction information. canEvolve allows querying of results of primary analysis, integrative analysis and network analysis of oncogenomics data. The querying for primary analysis includes differential gene and miRNA expression as well as changes in gene copy number measured with SNP microarrays. canEvolve provides results of integrative analysis of gene expression profiles with copy number alterations and with miRNA profiles as well as generalized integrative analysis using gene set enrichment analysis. The network analysis capability includes storage and visualization of gene co-expression, inferred gene regulatory networks and protein-protein interaction information. Finally, canEvolve provides correlations between gene expression and clinical outcomes in terms of univariate survival analysis.

**Conclusion:**

At present canEvolve provides different types of information extracted from 90 cancer genomics studies comprising of more than 10,000 patients. The presence of multiple data types, novel integrative analysis for identifying regulators of oncogenesis, network analysis and ability to query gene lists/pathways are distinctive features of canEvolve. canEvolve will facilitate integrative and meta-analysis of oncogenomics datasets.

**Availability:**

The canEvolve web portal is available at http://www.canevolve.org/.

## Introduction

At the 10^th^ anniversary of the human genome, high throughput experimental data explosion fueled by various functional genomics technologies is expected to overwhelm genomics data analysis [1]. This explosion is most evident in oncogenomics, where a vast number of tumors profiled by individual laboratories, together with data from large-scale projects such as the Cancer Genome Atlas (TCGA) [2] and the International Cancer Genome Consortium [3] is overwhelming the researchers. On the positive side, this data deluge has the potential to allow cancer researchers to address the second grand challenge outlined by Collins *et al*. [4]: translating genome-based knowledge into human health benefit. Meta-analysis and integrative analysis of these data and dissemination of results are essential for the scientific community engaged in basic cancer biology and translational research.

A few analysis questions frequently arise from the quest of extracting meaningful knowledge from oncogenomic profiles. For example, is the expression of my gene or miRNA of interest significantly altered in a cancer type compared to normal tissue? Is the copy number of my gene of interest altered in a cancer type? Can the expression changes of genes or proteins explained by underlying copy number alterations (CNAs) and mutations? Which genes and alterations are regulators of tumorigenesis? What are the genes whose expression changes have prognostic implications in a given tumor type? Which pathways or modules change their overall expression, and which functional categories are enriched above chance in altered genes?

A web portal that allows researchers to query results of different types of analysis with a view to generate novel hypotheses is an ideal platform for obtaining and disseminating such knowledge. However, generating such a portal is a challenging task. The tumor profiles have been generated in different laboratories using a variety of functional genomics platforms. They harbor “noise” from experimental variation along with true biological variation, and lack consistent annotations. Expert knowledge in oncology is required to frame appropriate analysis questions. Understanding of statistics and machine learning is required to select appropriate methodology for pre-processing, normalizing and integrating these data. Our recent work suggests that methods for integrating diverse data types are still evolving and face unique challenges due to ultra-high dimensionality of oncogenomic data [5]. Finally, knowledge of procedural, statistical and web programming is required to establish analysis pipelines and build user-friendly web interface. There are several databases that store and provide knowledge from oncogenomic profiles. GEO [6], [7] and ArrayExpress [8] are large public repositories of functional genomics datasets that include oncogenomic profiles. Although there have been some attempts to organize these data in resources such as Oncomine [9] and Genevestigator [10], both focus on analyses of limited data types and neither fully addresses the problem of integration across multiple data types generated from the same patients.

To address these challenges, we have developed the canEvolve web portal with the following aims. The portal should store functional genomics and other large-scale data on cancer. This includes gene and miRNA expression profiles, and copy number changes. The portal should provide stored knowledge in database as well as generate analysis results from oncogenomic profiles in response to user queries. This includes primary, integrative and network analysis of oncogenomic profiles. It should allow visualization of knowledge and analysis results in an appropriate manner and let the user download query results and related information from the portal. Finally, it should let the user compare multiple datasets. We have designed the canEvolve query functionalities to fulfill most frequent analysis requirements of cancer researchers towards generating novel biological hypotheses.

## System and Methods

### canEvolve architecture and data storage capabilities

The canEvolve web portal is implemented using mySQL open source system. The schema includes 44 tables divided into multiple modules (Figure S1 and Figure S2). The database can store information derived from functional genomics profiles from microarray and next generation sequencing platforms downloaded from GEO [6], [7]. Specifically, it stores normalized data in which experimental variation has been removed, and data on which primary and higher order analysis has been carried out. The processed data and analysis results stored at the portal include differential gene expression, differential miRNA expression, protein expression, copy number alterations and survival analysis. The network-based data stored at the portal include gene co-expression clusters, regulatory network clusters and protein-protein interactions. Integrative analysis results include gene set enrichment analysis (GSEA) [11] and integrative analysis of gene expression profiles with copy number alterations [12] and miRNA profiles [13]. Finally, canEvolve also stores thousands of human protein-protein interactions from STRING [14], 287 transcription factor-gene target information derived from TRANSFAC [15] and 885 miRNA-gene target information derived from PICTAR [16] The canEvolve web interface is implemented using Javascript and PHP.

### Software packages used for generating data analysis pipelines

The majority of analysis framework is written in the R programming language utilizing Bioconductor [17] modules and other open source packages. The genomics profiling datasets processed by the canEvolve pipeline have been curated from published studies. Thus, the selected datasets are already publication quality. They are processed and normalized using standard analysis methods. Specifically, microarray data and associated annotations are downloaded using the GEOquery package [18]. The Bioconductor affy [19] and simpleaffy packages are used to pre-process and normalize the data. Raw data (CEL) files from experiments run on the Affymetrix GeneChip platform are processed with the RMA normalization in the ‘affy’ package for each experimental group (study). For each GeneChip platform, probe set definition and other annotations are obtained from chip description files (CDF) supplied by Affymetrix, and sample information accompanying genomic profiles is parsed and manually curated. Normalization of miRNA studies is done in a similar fashion. LIMMA R package is used to identify differential expression [20]. Copy number profiling data are processed as described in Cao et al. [21]. The TCGA data incorporated into canEvolve are downloaded from Broad Institute's Genome Data Analysis Center (GDAC) at https://confluence.broadinstitute.org/display/GDAC/Home. For the TCGA data, the RNA-Seq data are normalized using the RSEM algorithm [22], thresholded copy number information is identified using GISTIC 2.0 [23], and protein expression data are normalized using SuperCurve method [24] by the Broad GDAC.

The MSigDB 3.0 curated gene sets are used to run Gene Set Enrichment Analysis [11]. The WGCNA [25] package is used to identify unsigned gene co-expression modules and the ARACNE [26] algorithm is used to infer regulatory networks from microarray data. A manually curated list of 2000 transcription factors (Shah PK et al., unpublished) is used as input for ARACNE. The list was generated using protein domain annotations from InterPro [27], gene ontology terms and literature searches. The DR-Integrator [12] package is used for integrative analysis of gene expression profiles with copy number alterations. The GemiNI [28] method is used for integrative analysis of gene expression profiles with miRNA profiles. Multiple hypothesis testing is adjusted using Benjamini-Hochberg correction as implemented in multtest R package [29]. We have utilized many of these pipelines in the past and compared our results to published studies, and have found that these pipelines are error free and generate reproducible results for each analysis type.

## Results

### canEvolve database content

The canEvolve web portal 1.0 is available at http://www.canevolve.org. It is designed to answer primary and integrative analysis questions frequently asked by cancer biologists. The current version provides different types of information extracted from 90 studies profiling more than 10,000 patients (Table 1), including 15 TCGA datasets containing 4800 patient profiles. In addition to information on differential gene and miRNA expression and changes in gene copy number, it stores hundreds of thousands of instances of co-expression, protein-protein interaction, and metabolic and signaling pathways for the human proteome. It also stores transcription factor-target and miRNA-target information. The number of different analysis types for different cancer types is summarized in Table 2 and Figure S3. We are continuously adding new datasets of various cancer types into canEvolve and the updated information is at the “About/Statistics” section of the portal.

**Table 1:** The number of datasets for different data types in canEvolve.

Data Type	Total Datasets	Total Patients
Gene Expression	55	6677
Copy Number Alterations	43	6537
miRNA Expression	7	466
Mutation	14	2867
Protein Expression	8	2190
Protein-Protein Interactions	NA	NA

**Table 2:** Data analysis algorithms and total analyzed datasets in canEvolve.

Analysis type	Analysis method	Software/algorithm	Analyzed datasets
Primary	Differential Gene Expression	LIMMA	68
Primary	Differential miRNA Expression	LIMMA	19
Primary	Copy Number Alterations	dChipSNP	32
Network	Regulatory Networks	ARACNE	13
Network	Co-expression Networks	WGCNA	16
Integrative	Gene Set Enrichment	GSEA	16
Integrative	Gene Expression and miRNA Integration	GemiNI	6
Integrative	Gene Expression and Copy Number Alterations	DR-Integrator	6
Integrative	Genomic Changes and Gene Expression	RSEM/GISTIC 2.0	14
Integrative	Genomic Changes and Protein Expression	SuperCurve/GISTIC 2.0	8
Survival	Survival analysis	R package Survival	22

Literature references to the analysis algorithms are provided in the main text.

### canEvolve web interface

**Figure 1:**
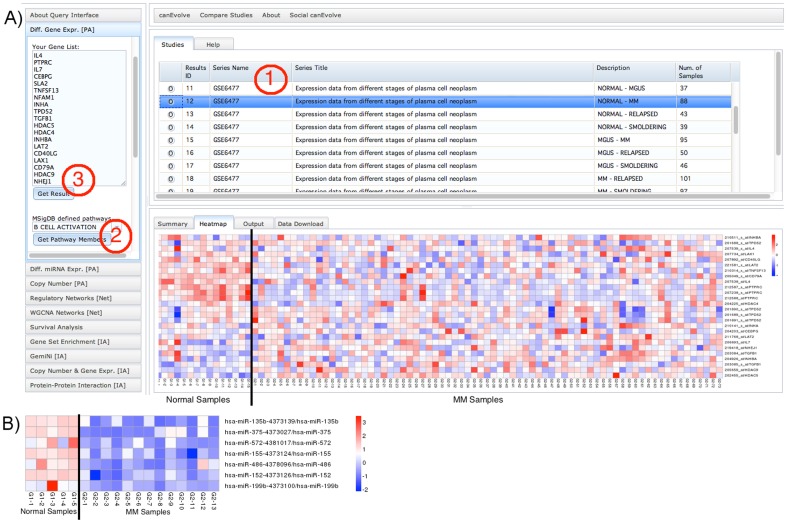
Query interface and visualization of primary analysis. (A) Visualization of differential gene expression for B-Cell Activation pathway members in normal versus multiple myeloma (MM) comparison using the GSE6477 data. (B) Heatmap of differential miRNA expression in normal versus MM comparison using the GSE16558 data. The MM samples are a subset that has no cytological abnormalities.

The canEvolve web interface is designed to be simple and uniform for querying different types of analysis. The query page at http://www.canevolve.org/ lets a user retrieve the stored knowledge and analysis results in easy steps (Figure 1A). First, the user selects an analysis type at the left panel. Second, the user selects a cancer type and studies stored in the database. Third, the user inputs a gene name, a list of genes or select pathways, and clicks “Get Results” to query the database and obtain results. The query interface accepts official gene symbols. Depending on the analysis type, query results can be visualized as heatmaps (Figure 1A), plots or networks. The ‘Help’ tab located at the top of the query page provides step-by-step instructions to effectively use canEvolve. The query results can also be downloaded in the form of tables and R data objects.

In the following we show examples of canEvolve capabilities and how the stored knowledge and analysis results can be useful for cancer researchers to generate biological hypotheses. We take examples of genes and gene sets that may play important roles in pathogenesis of multiple myeloma (MM) [30] and lung cancer [31].

### Examples of canEvolve query and visualization

The primary analysis capabilities include abilities to query differential gene and miRNA expression as well as changes in copy numbers (Figure 1). As a response to user queries canEvolve portal creates an output page with four tabs providing query summary, visualization, tabular data output and a data download option. The gene set “B Cell Activation Pathway” as defined by MSigDB version 3 [11] was used to generate Figure 1A, and a list of 7 miRNAs was used to generate Figures 1B.

**Figure 2:**
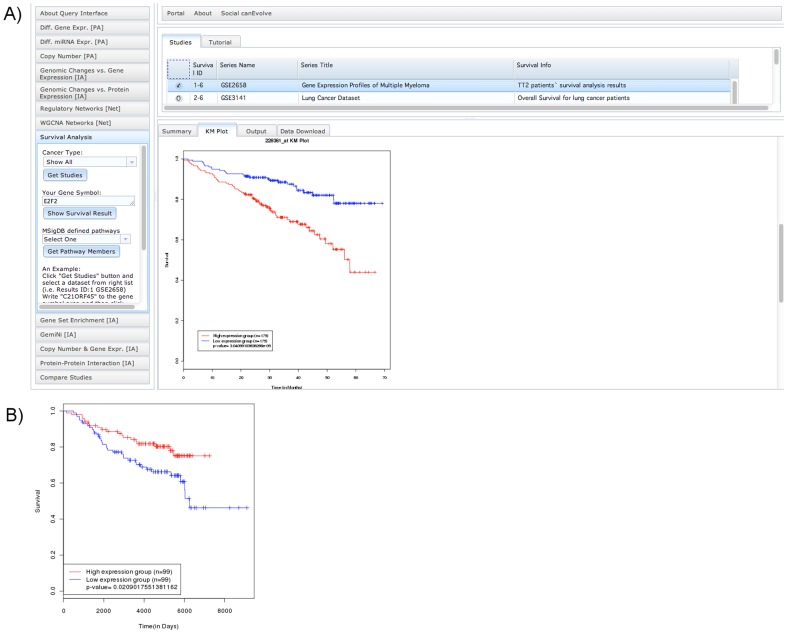
Query interface and visualization of survival analysis. (A) The Kaplan-Meier plot on the lower-right shows the survival impact of E2F2 gene expression in the multiple myeloma dataset GSE2658. Two expression groups are defined using the median E2F2 gene expression across all samples as the splitting value, and log-rank test is used to compute the p-value. Higher expression of E2F2 leads to high-risk (red) while lower expression leads to lower risk (blue).

The “Survival analysis” module carries out a univariate survival analysis, showing that the gene expression of transcription factor (TF) E2F2 significantly correlates with overall patient survival in MM (Figure 2A), and the gene expression of MAP2K4 significantly correlates with overall survival in breast cancer (Figure 2B). These two genes are involved in cell cycle checkpoint and signaling transduction pathways, respectively, and their correlation with survival outcomes suggests their roles in pathogenetic pathways and potential as prognosis markers.

**Figure 3:**
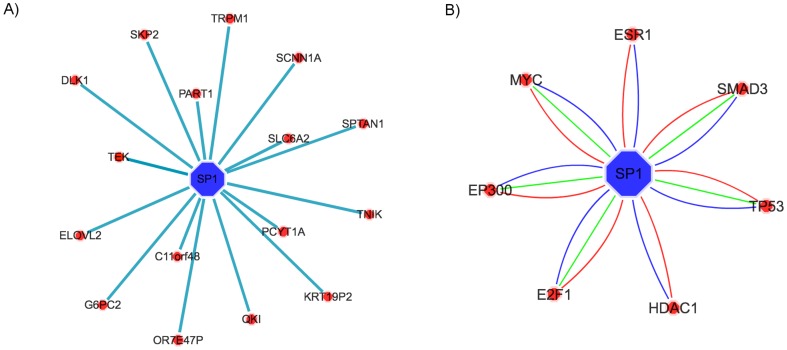
Network visualization by interfacing Cytoscape from canEvolve. (A) ARACNE reconstructed gene regulatory network for the transcription factor SP1 using the multiple myeloma dataset GSE6477. (B) Experimentally validated and predicted Human protein-protein interaction network of SP1 derived from the STRING database at the threshold of 0.993. The three lines connecting SP1 to different proteins show distinct evidence types as used by STRING.

The TF SP1 [32] is used to query and visualize ARACNE reconstructed transcriptional regulatory network in MM (Figure 3A) and human protein-protein interaction network for the gene (Figure 3B). These examples also show the ability of canEvolve portal to generate high quality images.

**Figure 4:**
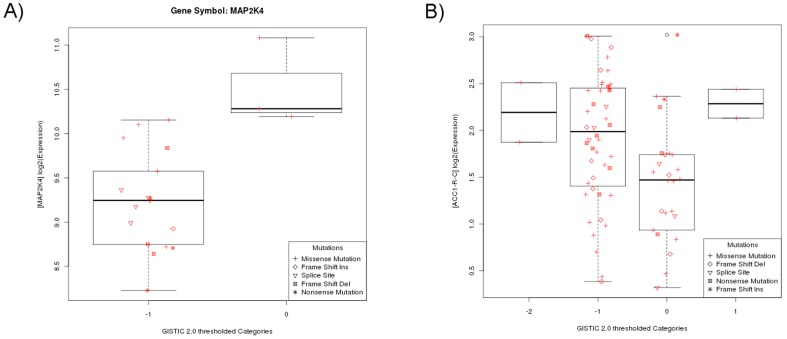
Visualization of the association between genomic abnormality and gene or protein expression. (A) Boxplots of the expression of gene MAP2K4 (X-axis) is plotted against groups of samples with different levels of copy number alteration of the MAP2K4 gene (Y-axis). Different mutation types of the BRCA gene in these samples are also indicated. (B) Similar to (A), but Y-axis represents the protein expression of gene ACC1, and X-axis and mutation points are represent the copy number abnormalities and mutation of TP53. Both (A) and (B) use the TCGA LUAD dataset.

The portal allows users to inspect the association between genomic abnormalities and gene or protein expression levels for TCGA patient profiles (Figure 4). This is accomplished by visualizing the relationship between copy number alterations (X-axis of Figure 4), gene expression levels (Y-axis) and mutations of the same gene or of two different genes across patients. Moreover, canEvolve provides opportunity to integrate information derived from TCGA profiles to the publicly available profiles. For example, users can infer the differential gene expression (Figure 4A) and survival impact (Figure S4) of MAP2K4 differential gene expression in breast cancer using information from TCGA and GSE7390.

### Integrative analysis capabilities

The canEvolve portal allows researchers to query and retrieve results from different types of integrative analysis. The simplest integrative analysis is the ability to query differential expression and survival impact of mSigDB curated gene sets (Figure 1A). The canEvolve also provides pre-calculated GSEA results that allows integration of gene expression information with mSigDB curated gene sets, such as chromosome-position based gene sets, computationally identified gene sets that share a cis-regulatory motif, or gene ontology terms.

**Figure 5:**
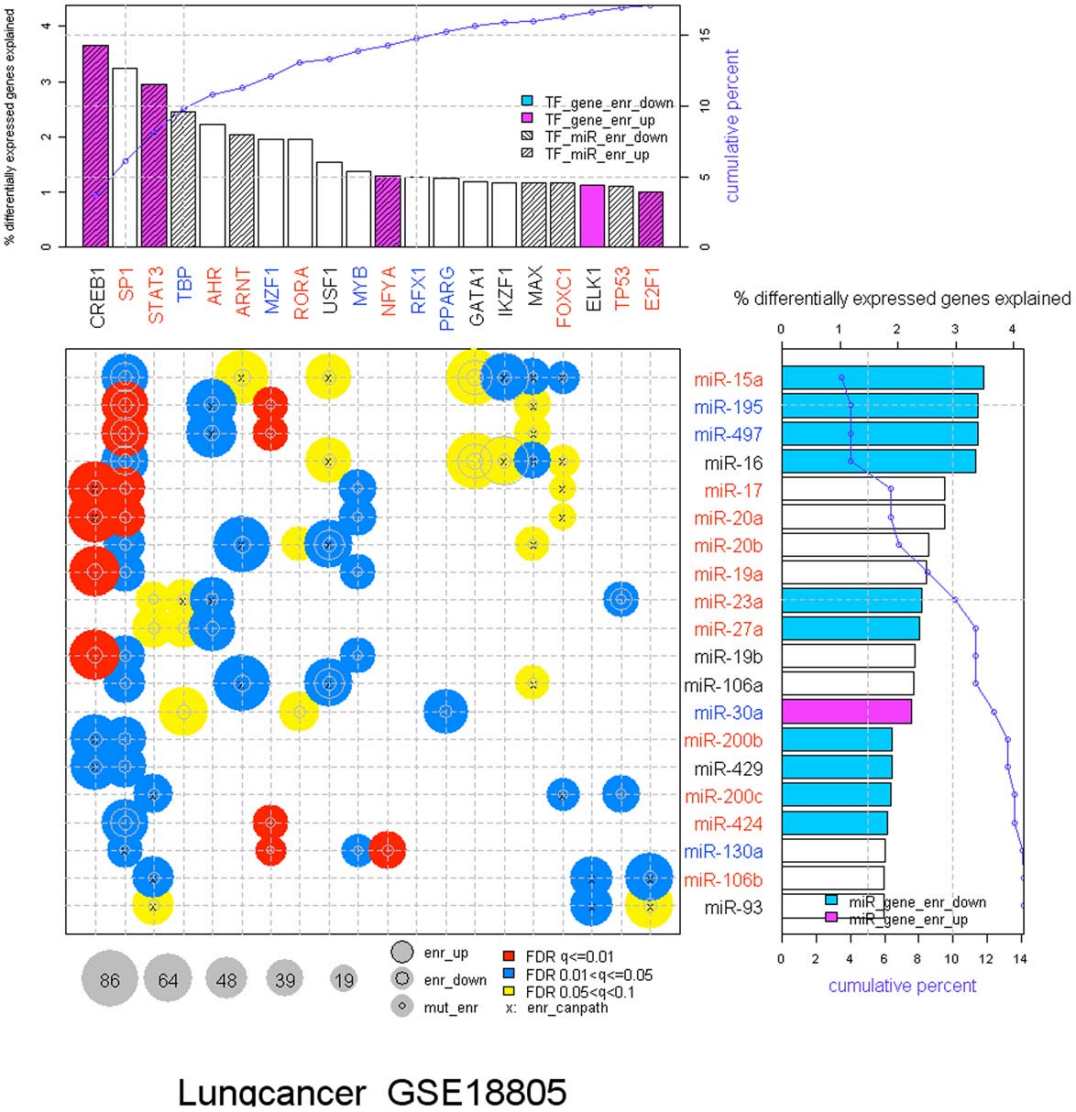
dChip-GemiNI analysis integrating gene expression with miRNA expression. The summary bubble-bar plot from GemiNI analysis using the lung cancer dataset GSE18805 to identifies candidate transcription factors, miRNAs, and TF-miRNA feed-forward loops (FFL) involved in cancer pathogenesis. TFs and miRNAs are ranked by the percentage of normal-cancer differentially expressed genes explained by all the significant FFLs involving a TF or miRNA (the height of bars). The top 20 TFs and miRNAs are displayed. The bubble size indicates the number of differentially expressed FFL target genes, and color indicates the FFL significance. For more details on the figure and the methodology see [28].

The canEvolve portal also identifies genes that are putative drivers or regulators tumorigenesis. We recently reported the GemiNI (Gene and miRNA Network-based Integration) method for integrating gene and miRNA expression profiles using feed-forward loops consisting of TFs, miRNAs and their common target genes [28]. GemiNI-identified TF and miRNAs regulators are available for query at the canEvolve portal (Figure 5). For example, GemiNI analysis of a lung cancer data set with paired gene/miRNA expression (GSE18805, [33]) identified top TFs (CREB1, SP1 and STAT3) and miRNAs (miR-15a, miR-195 and miR-497) that are dysregulated in lung cancer. These TFs and miRNAs have either known roles in lung cancer and other cancer types or are potential new targets for experimental validation [34] [35] [36], [37].

In addition, researchers can access gene sets with highly concordant gene expression changes and copy number alterations based on DR-Integrator analysis [12]. These genes are likely to be enriched of oncogenes and tumor suppressor genes [38]. For example, BIRC2 and FAF1 are among the top 10 genes identified using DR-Integrator analysis of a paired copy number and gene expression dataset for myeloma (Table 3) [39]. These genes have also been found to be often homozygous deleted and with survival impact for myeloma by another independent study [40].

**Table 3:** Top 10 genes identified from integrative analysis of copy number profiles with gene expression profiles from the multiple myeloma dataset GSE26863 [39].

Gene Symbol	Rank	Gene/copy Correlation	FDR
BIRC2	1	0.8666	0
PSMD4	2	0.7784	0
SDHC	3	0.7614	0
UBAP2L	4	0.75	0
MRPL9	5	0.7386	0
JTB	6	0.736	0
FAF1	7	0.7358	0
GPR89A	8	0.7352	0
WHSC1L1	9	0.7351	0
GSTT1	10	0.7346	0

### Meta-analysis of multiple studies

**Figure 6:**
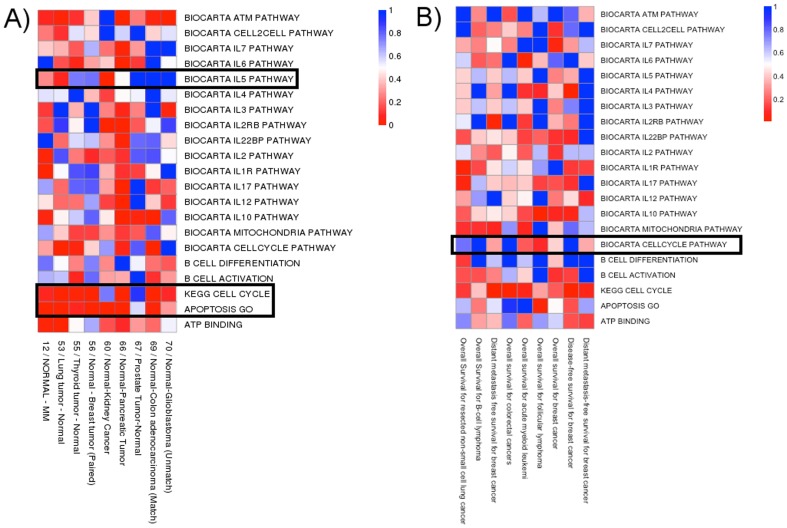
Meta-analysis of multiple studies in canEvolve. (A) The colors in the heatmap show the Fisher's Exact test p-value for the enrichment of differentially expressed genes between normal-cancer comparisons (X-axis) in a KEGG or Biocarta pathway (Y-axis). (B) Similar to (A), but gene sets on the X-axis are selected for their significant correlation with survival using the cox proportional hazards model.

The canEvolve “Compare Studies” function allows meta-analysis of pathways across multiple studies for differentially expressed genes. The function allows users to select multiple studies and check the enrichment of MSigDB derived gene sets in differentially expressed or survival-related genes from these studies. Figure 6A shows such a comparison for 21 gene sets across 9 different cancer types. The figure suggests that pathways such as cell cycle and apoptosis are more commonly dysregulated across multiple cancer types, while the dysregulation of other pathways such as IL5 is cancer type specific. Similarly, Figure 6B reveals pathways that have survival correlations only in specific cancer types, such as the cell cycle pathway in breast cancer but not non-small cell lung cancer [41].

### Identifying putative regulators of multiple myeloma evolution

Meta-analysis of multiple studies not only provides insights into differential pathway utilization or prognosis but also allows us to model the evolution of different cancer types and candidate regulators responsible for the process. At present, such analysis is difficult due to the lack of suitable functional genomics profiles covering all the stages of cancer evolution from the same patients. Here we provide modeling of myeloma (MM) evolution as an example of mining GSEA results stored in canEvolve. MM evolves from a pre-malignant stage called monoclonal gammopathy of undetermined significance (MGUS) at the rate of 1% per year [42]. With response rate of about 40% with individual drugs, many treated MM patients relapse. Currently, little is known about this process of MM evolution [43], [44], specifically about the changes in regulatory networks and signaling pathways responsible for it.

**Figure 7:**
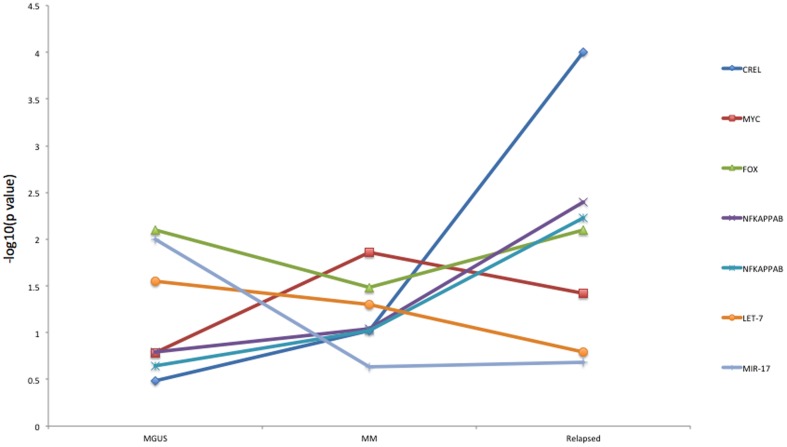
Modeling of multiple myeloma evolution. Identification of transcription factors and miRNAs whose target genes significantly change their overall expression compared to normal plasma cells during the evolution of myeloma from MGUS to relapsed stages by gene set enrichment analysis. The X-axis shows different evolutionary stages of myeloma. The Y-axis shows the –log10 (p-value) from the gene set enrichment analysis using the target genes of a TF or a miRNA based on MSigDB.

To model the evolution of MM with canEvolve, we carried out gene set enrichment analysis of normal-MGUS, normal-MM, normal-relapsed MM [45], with regulatory and pathways gene sets from MSigDB (Figure 7). We identified transcription factors, miRNA, metabolic and signaling pathways whose targets/members significantly change their overall expression compared to normal plasma cells at different stages of cancer progression. For example, the targets of MYC, FOXO, NF-κB [46], miR-17 [47] and let-7 [48] family members significantly change expression as MGUS turns to MM. In contrast, the targets of miR-484 and CREL (a member of the NF-κB family, [49]) significantly change as MM patients relapse. These results suggest experimental directions that target cancer evolution for therapeutics.

## Discussion

We have created the canEvolve portal to help cancer biologists easily access the knowledge and analysis results derived from primary, integrative and network analysis of oncogenomic data generated using various functional genomics platforms. The algorithms for the analysis pipelines are selected from our experiences in creating and utilizing such tools for generating biologically relevant hypotheses. The focus of this work is the generation of the database framework capable of storing multiple data types and the user-friendly web interface.

The portal functionalities are developed with the analysis requirements and feedback from multiple myeloma researchers. We have now standardized those requirements and developed rules for selecting and analyzing datasets for different cancer types from public repositories to be added into canEvolve. canEvolve is currently actively being used for research and has had more than 150 unique visitors from 15 different countries and some of them have provided important feedback. Users can contact us at helpcanevolve.org for help, feature suggestions and dataset requests, or follow us on Facebook and Twitter.

Several existing databases and web portals allow researchers to query oncogenomic data. Most of them focus only on one data type (e.g. GCOD [50], CaSNP [21] and PrognoScan [51]). canEvolve allows users to query larger number of data types when suitable. It also allows visualization of regulatory and protein-protein interaction networks. The recently published cBio cancer genomics portal [52] allows access to level 3 TCGA data from the Broad Institute's genome data analysis center and provides query capabilities similar to canEvolve. Unlike the cBio portal, canEvolve provides higher-level analysis and allows users to integrate TCGA data with other publicly available data. The research edition of Oncomine provides standard analysis such as comparison of cancer vs. normal, multi-cancer analysis, co-expression, cancer outlier profile analysis and molecular concept map analysis. Other Oncomine functionalities require subscription. Unlike Oncomine, all canEvolve functionalities are available for free. Moreover, neither the cBio portal nor Oncomine provides network-based as well as integrative analysis of multiple data types provided by canEvolve. While the canEvolve query functionalities are general-purposed, the choice of analysis algorithms (e.g. ARACNE, GemiNI) makes canEvolve a useful tool to extract inference on regulators of gene expression such as transcription factors and miRNAs. Also, canEvolve facilitates pathway-level inference of abnormal gene expression and copy number changes, and their survival impact. None of the existing portals have such focus.

At present, many canEvolve processing and visualization functions compute in real time. This design decision has resulted in a substantial savings of disk space but it has slowed the response time to user queries. This will be remedied in the next version of canEvolve that will be based on cloud computing. Cloud computing can accelerate the processing time by providing on-demand resources for queries and Hadoop-based distributed computing for running analysis. Currently we are redesigning some of the processing and visualization pipelines to use R with the Hadoop framework. The next version of canEvolve will better integrate regulatory and protein-protein interaction information. It will also allow researchers to analyze their own datasets in light of current knowledge, stored analysis results and state-of-the-art methodologies available at the portal in the form of automated workflows. Finally, we will regularly insert level 3 TCGA data and develop functions for further analysis of these data.

## Supporting Information

Figure S1 **Overall organization of canEvolve.**
(TIF)

Figure S2 **Modules in the canEvolve database schema.**
(TIF)

Figure S3 **Number of data sets, comparisons for different analysis types for different cancer types in canEvolve.**
(TIF)

Figure S4 **Survival curves for MAP2K4. See** **figure 4** **legend for more information.**
(TIF)
