# Supplementary figures and images for: canEvolve: A Web Portal for Integrative Oncogenomics
Source: PLoS One. 2013 Feb 13;8(2):e56228. doi: 10.1371/journal.pone.0056228 (PMC3572035; doi:10.1371/journal.pone.0056228)

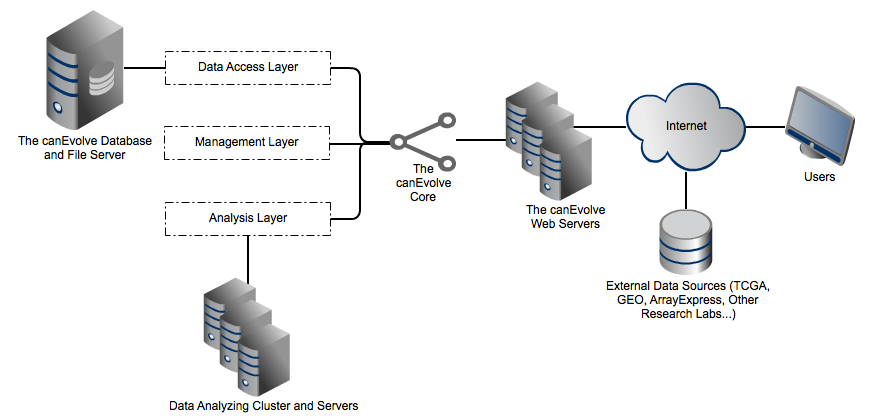

Supplement: Figure S1 — Overall organization of canEvolve. (TIF) [file pone.0056228.s001.tif]

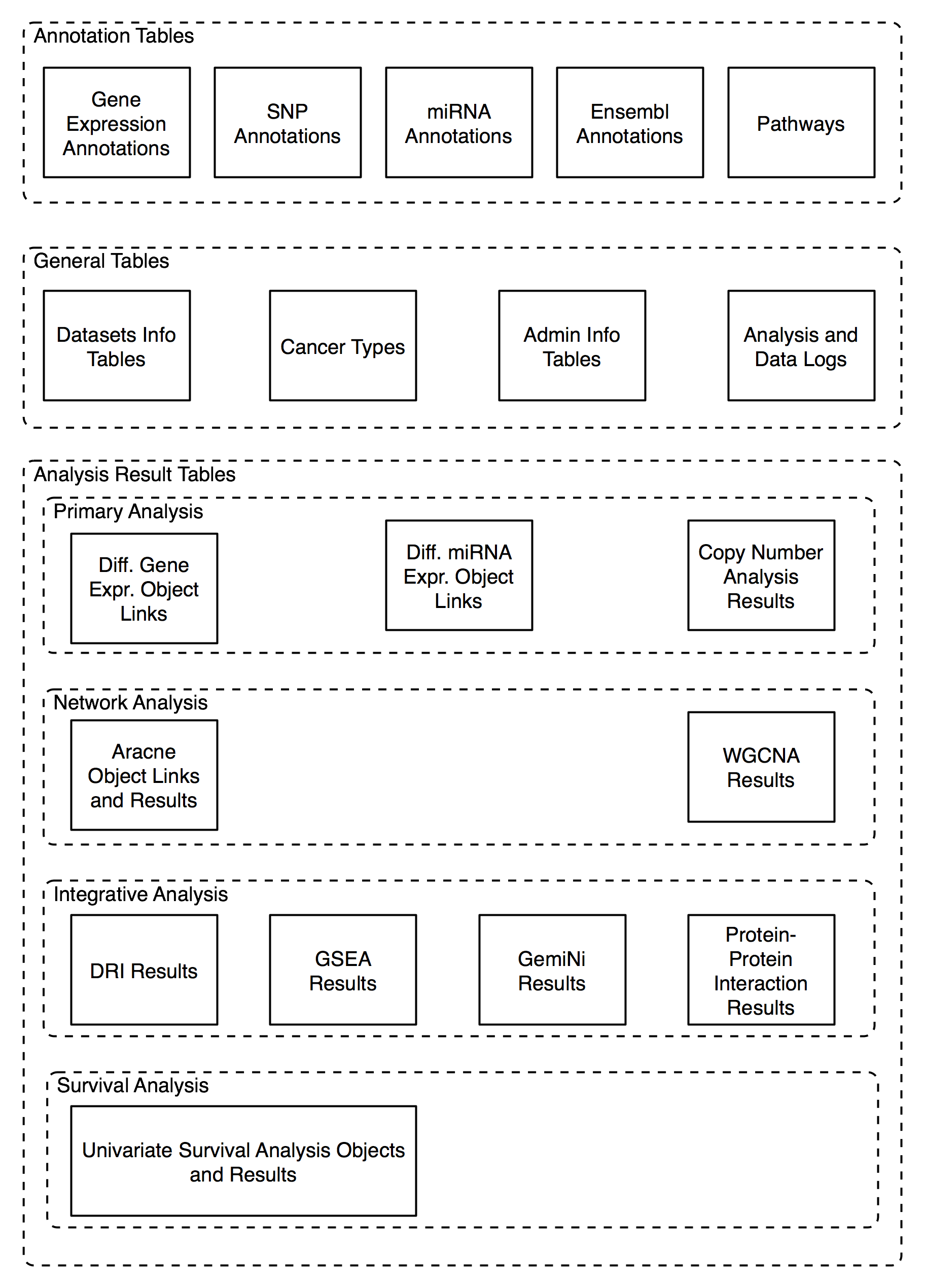

Supplement: Figure S2 — Modules in the canEvolve database schema. (TIF) [file pone.0056228.s002.tif]

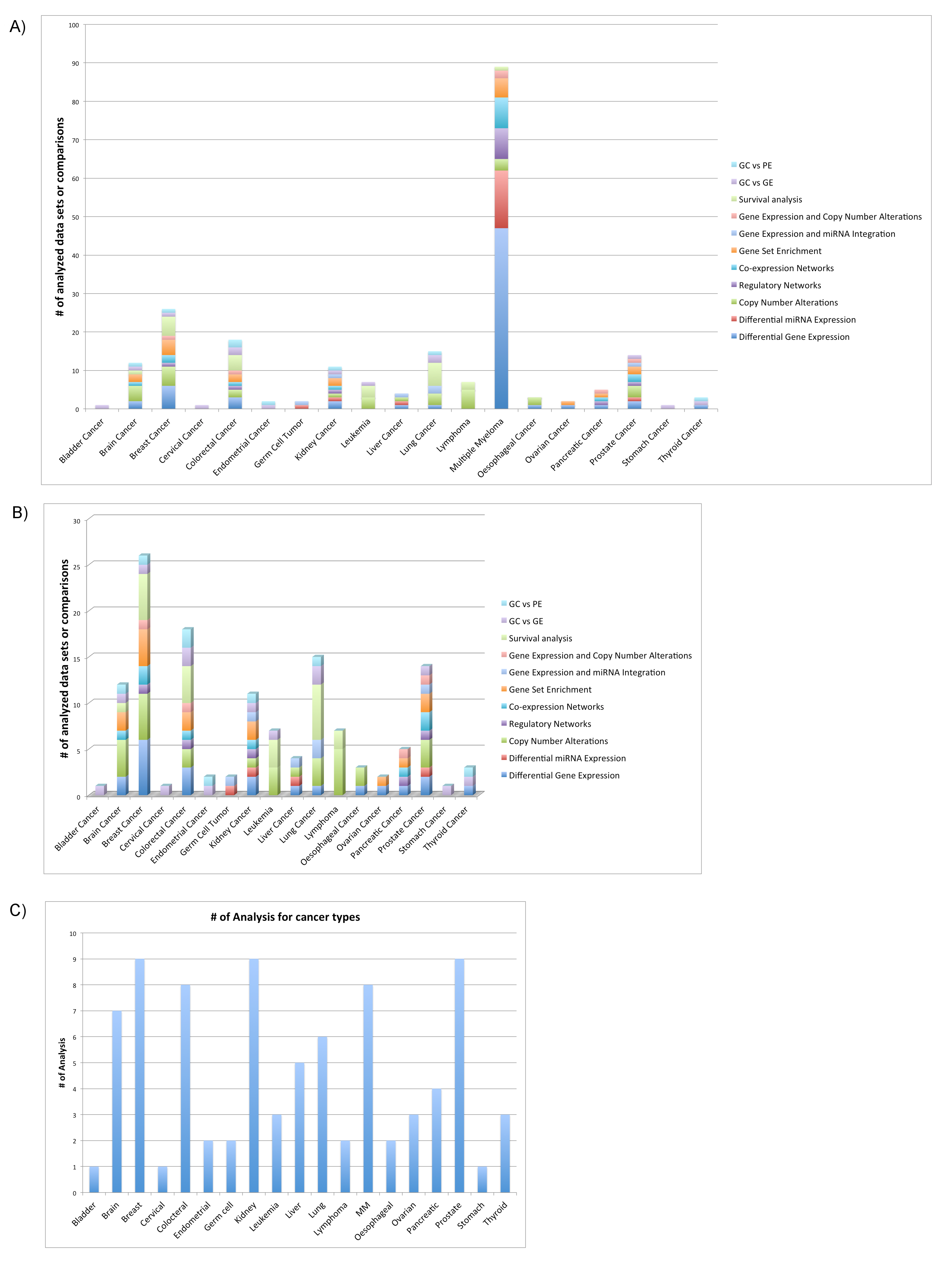

Supplement: Figure S3 — Number of data sets, comparisons for different analysis types for different cancer types in canEvolve. (TIF) [file pone.0056228.s003.tif]

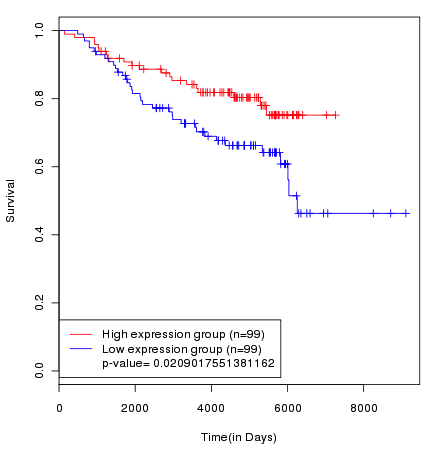

Supplement: Figure S4 — Survival curves for MAP2K4. See figure 4 legend for more information. (TIF) [file pone.0056228.s004.tif]
